# Patient perceptions of acute pain and activity disruption following inguinal hernia repair: a propensity-matched comparison of robotic-assisted, laparoscopic, and open approaches

**DOI:** 10.1007/s11701-018-0790-9

**Published:** 2018-02-16

**Authors:** James G. Bittner IV, Lawrence W. Cesnik, Thomas Kirwan, Laurie Wolf, Dongjing Guo

**Affiliations:** 1 Department of Surgery, Virginia Commonwealth University School of Medicine, PO Box 980519, Richmond, VA 23298 USA; 2 Market Research Department, Intuitive Surgical, Inc., Sunnyvale, CA USA; 3 Bruno and Ridgway Research Associates, Lawrenceville, NJ USA; 4 Department of Clinical Affairs, Intuitive Surgical, Inc., Sunnyvale, CA USA

**Keywords:** Inguinal hernia, Groin hernia, Inguinal hernia repair, Robotic, Robotic assisted, Robotic-assisted inguinal hernia repair, Laparoscopic inguinal hernia repair, Groin pain, Postoperative groin pain

## Abstract

Few publications describe the potential benefit of robotic-assisted inguinal hernia repair on acute postoperative groin pain (APGP). This study compared patients’ perceptions of APGP, activity limitation, and overall satisfaction after robotic-assisted- (R), laparoscopic (L), or open (O) inguinal hernia repair (IHR). Random samples of patients from two web-based research panels and surgical practices were screened for patients who underwent IHR between October 28, 2015 and November 1, 2016. Qualified patients were surveyed to assess perceived APGP at 1 week postoperatively, activity disruption, and overall satisfaction. Three cohorts based on operative approach were compared after propensity matching. Propensity scoring resulted in 83 R-IHR matched to 83 L-IHR respondents, while 85 R-IHR matched with 85 O-IHR respondents. R-IHR respondents recalled less APGP compared to respondents who had O-IHR (4.1 ± 0.3 vs 5.6 ± 0.3, *p* < 0.01) but similar APGP compared to L-IHR (4.0 ± 0.3 vs 4.4 ± 0.3, *p* = 0.37). Respondents recalled less activity disruption 1 week postoperatively after R-IHR versus O-IHR (6.1 ± 0.3 vs. 7.3 ± 0.2, *p* < 0.01) but similar levels of activity disruption after R-IHR and L-IHR (6.0 ± 0.3 vs. 6.6 ± 0.27, *p* = 0.32). At the time of the survey, respondents perceived less physical activity disruption after R-IHR compared to O-IHR (1.4 ± 0.2 vs. 2.8 ± 0.4, *p* < 0.01) but similar between R-IHR and L-IHR (1.3 ± 0.2 vs 1.2 ± 0.2, *p* = 0.94). Most respondents felt satisfied with their outcome regardless of operative approach. Patient perceptions of pain and activity disruption differ by approach, suggesting a potential advantage of a minimally invasive technique over open for IHR. Further studies are warranted to determine long-term outcomes regarding pain and quality of life after IHR.

## Introduction

In the United States (US), an estimated 4.5 million people are impacted by groin hernia, and each year approximately 800,000 people are treated for groin hernia [1, 2]. Chronic postoperative groin pain (CPGP) is an infrequent but notable complication associated with inguinal hernia repair (IHR) [3]. Two important risk factors for CPGP are preoperative groin pain and acute postoperative groin pain (APGP) [4, 5]. APGP and CPGP can impact patient quality of life after IHR [6, 7], and the American Pain Society and American Society of Anesthesiologists developed guidelines to aid the management of these conditions [8].

One method to potentially lower the risk of APGP, and subsequently CPGP, is to adopt a minimally invasive approach to IHR [9]. Robotic-assisted inguinal hernia repair (R-IHR) is a minimally invasive approach increasing in frequency in the US [10]. Despite the known advantages of laparoscopic inguinal hernia repair (L-IHR), including less early postoperative pain, shortened hospital stay, and less wound infections [11], few publications have investigated the potential benefit of R-IHR on APGP [12]. To that end, the present study compared three surgical approaches—open (O), laparoscopic (L), robotic-assisted (R) IHR—and the associated patient perceptions of APGP, activity limitations, and overall satisfaction.

## Materials and methods

### Study population

Respondents were included in the study if they underwent O-IHR, L-IHR, or R-IHR between October 28, 2015 and November 1, 2016 and completed the survey in its entirety. Individuals were excluded from the study if they did not reply to or refused the invitation to participate; did not self-identify as having undergone IHR; reported IHR before October 28, 2015; failed to complete the survey; or represented duplicate data (e.g., digital fingerprinting indicated the participant was a panelist in both market research databases).

### Data source and collection

A random sample of consumers from two web-based research panels was screened to participate in the study. Invitations were sent by the market research companies, Precision Sample (Denver, Colorado) and Survey Sampling International (Shelton, Connecticut). Additional potential panelists were contacted via surgical practice outreach. Individuals who met the screening criteria and expressed interest in the study were sent an email invitation to participate in a HIPAA compliant survey via Survey Writer (Chicago, IL, USA). Respondents were incentivized $5.00 or $10.00 US upon completion of the survey, which required approximately 10 min of time. Up to two email reminders were sent to individuals who expressed interest to participate but did not initiate or complete the survey. Respondents had the opportunity to opt out of the survey at any time. Survey data from respondents were collected from October 25, 2016 to December 2, 2016.

Respondents were asked to rate their hernia-related groin pain 1 week preoperatively, APGP at 1 week postoperatively (primary measure), and APGP at the time of survey completion (0–10 scale, based on the validated Numeric Pain Rating Scale) [13]. Respondents also rated perceived activity disruption 1 week postoperatively and at the time of survey completion, as well as overall satisfaction with IHR (0–10 scales, modified from the Numeric Pain Rating Scale). Other survey questions inquired as to a history of IHR, regular use of preoperative pain medications, length of time from operation to resolution of APGP, duration of prescription pain medications, and return to normal/unrestricted physical activity and full-duty work. The survey concluded with a series of demographic questions (age, gender, employment type, education level, and annual income).

### Analysis

Three respondent cohorts were created based on type of most recent IHR (open, laparoscopic, and robotic-assisted), and comparisons were made for R-IHR vs. O-IHR and R-IHR vs. L-IHR. Propensity scores were calculated using a logistic regression model with the following covariates: age category, education level, employment status, type of job/labor, income level, use of prescription pain medication prior to IHR (yes/no), and history of IHR (yes/no). Propensity score matching was performed with a caliper width of 0.05 using a greedy match method. Participants undergoing R-IHR were separately matched 1:1 to patients undergoing O-IHR and L-IHR, respectively. Continuous variables were summarized as mean ± standard error (SE). Comparisons between matched cohorts were performed using a Wilcoxon rank sum test for continuous variables and a Chi-square or Fisher’s exact test for categorical variables as appropriate. A *p* value less than 0.05 was considered statistically significant. All analyses were performed using SAS version 9.4 (SAS Institute, Inc., Cary, NC, USA).

## Results

**Fig. 1:**
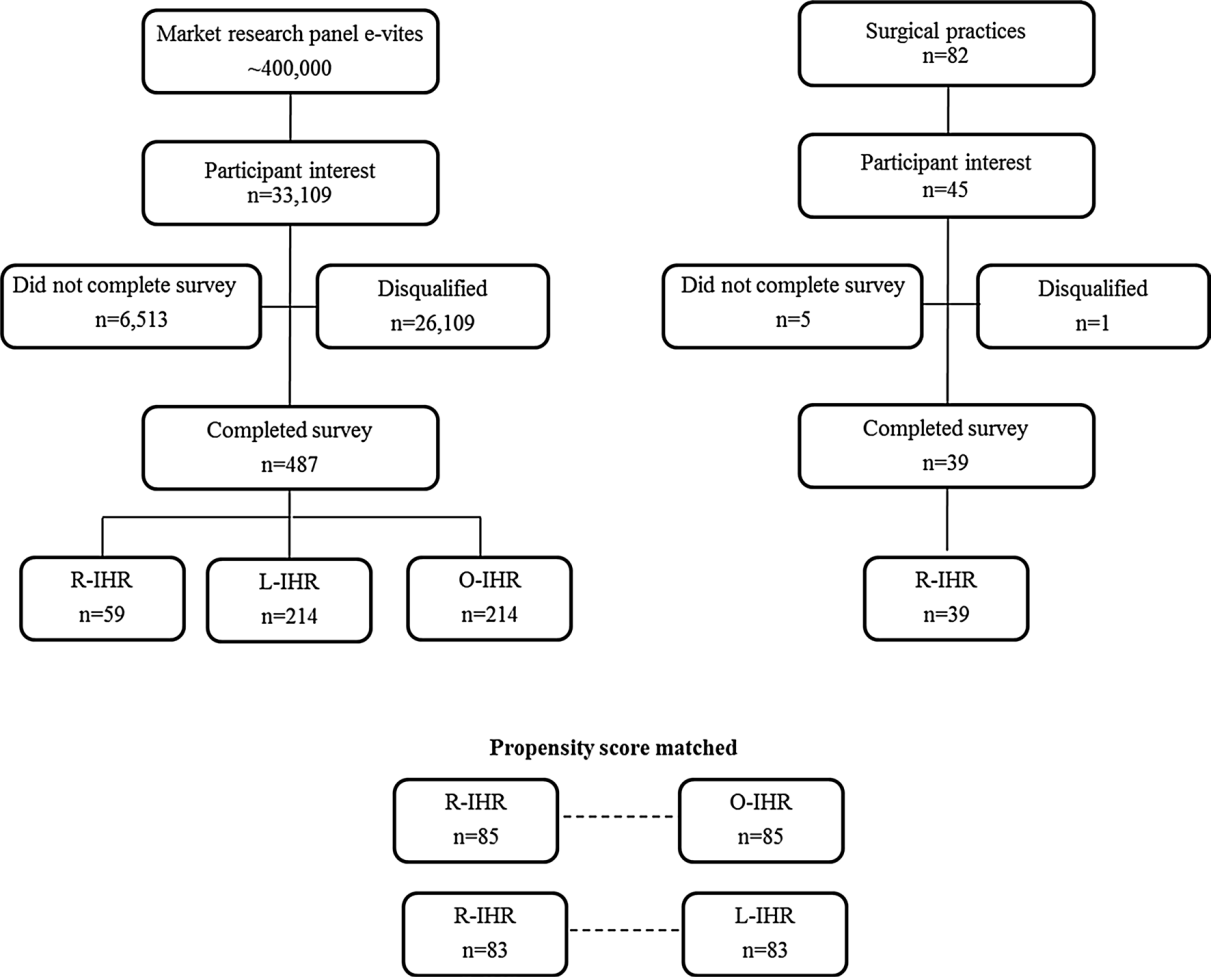
Survey respondent attrition and propensity-matched cohorts are shown

More than 33,100 individuals expressed interest to participate in the survey study with a 6% response rate. A total of 526 individuals met eligibility criteria for the study (214 O-IHR, 214 L-IHR, and 98 R-IHR). Propensity scoring resulted in 83 R-IHR matched to 83 L-IHR respondents, while 85 R-IHR were matched with 85 O-IHR respondents (Fig. 1).

### Demographics and clinical characteristics

Respondents were geographically dispersed across the US; 21% in the Northeast, 22% in the Midwest; 34% in the South, and 23% in the West, similar to proportions reported by the US Census Bureau in 2016. Prior to propensity matching, the cohorts differed by age, type of job labor, history of IHR, and use of preoperative pain medication. After matching, there were no significant differences between the cohorts among these demographic or clinical characteristics (Table 1).

**Table 1:** Demographics and clinical characteristics (propensity matched)

Characteristics	Robotic vs laparoscopic			Robotic vs open		
	R-IHR (*N* = 83)	L-IHR (*N* = 83)	*p* value	R-IHR (*N* = 85)	O-IHR (*N* = 85)	*p* value
Female, *n* (%)	2 (2.4)	0 (0.0)	0.15	2 (2.4)	1 (1.2)	0.56
Age, mean ± SD	54.4 ± 11.0	57.5 ± 12.3		53.2 ± 11.9	56.2 ± 12.0	
Education, *n* (%)						
Up to high school/GED	9 (10.8)	7 (8.4)	0.54	12 (14.1)	10 (11.8)	0.70
Some college	28 (33.7)	23 (27.7)		27 (31.8)	32 (37.6)	
College graduate or beyond	46 (55.4)	53 (63.9)		46 (54.1)	43 (50.6)	
Employment status, *n* (%)						
Full time	50 (60.2)	53 (63.9)	0.78	53 (62.4)	45 (52.9)	0.35
Part time	6 (7.2)	4 (4.8)		6 (7.1)	5 (5.9)	
Not employed	27 (32.5)	26 (31.3)		26 (30.6)	35 (41.2)	
Type of job labor, *n* (%)						
Heavy/very heavy labor	8 (9.6)	7 (8.4)	0.98	9 (10.6)	9 (10.6)	0.25
Light/medium labor	26 (31.3)	26 (31.3)		27 (21.8)	28 (32.9)	
No physical labor	22 (26.5)	24 (28.9)		23 (27.1)	13 (15.3)	
Not employed	27 (32.5)	26 (31.3)		26 (30.6)	35 (41.2)	
Using preoperative prescription pain medication, *n* (%)	25 (30.1)	21 (25.3)		27 (31.8)	25 (29.4)	
Prior history of inguinal hernia repair, *n* (%)	23 (27.7)	23 (27.7)		24 (28.2)	23 (27.1)	

*GED* general equivalency diploma, *IHR* inguinal hernia repair, *L* laparoscopic, *O* open, *R* robotic assisted, *SD* standard deviation

The *p* values were calculated using Wilcoxon rank sum test and a Chi-square or Fisher’s exact tests

### Postoperative groin pain

At 1 week postoperatively, R-IHR respondents recalled significantly less APGP compared to O-IHR (4.1 ± 0.3 vs 5.6 ± 0.3, *p* < 0.01) but a similar amount compared to the L-IHR cohort (4.0 ± 0.3 vs 4.4 ± 0.3, *p* = 0.37). Time to resolution of APGP did not significantly differ between cohorts. Respondents reported a shorter duration of prescription analgesic medication use after R-IHR compared to O-IHR (9.4 ± 1.4 vs. 10.6 ± 1.2 days, *p* = 0.03). While a trend existed such that patients who underwent R-IHR reported a shorter duration of prescription analgesic medication use compared to those after L-IHR, the difference was not significant (9.4 ± 1.5 vs. 11.6 ± 1.7 days, *p* = 0.30). A summary of postoperative pain measures appears in Table 2. At a mean follow-up of 6 months, similar proportions of respondents noted CPGP after R-IHR compared to O-IHR (50 vs. 57%, *p* = 0.52) and L-IHR (54 vs. 72%, *p* = 0.06).

**Table 2:** Time variables related to postoperative groin pain and activity level (propensity matched)

Measures	Robotic vs laparoscopic					Robotic vs open				
	R-IHR (*n* = 83)		L-IHR (*n* = 83)		*p* value	R-IHR (*n* = 85)		O-IHR (*n* = 85)		*p* value
	*n*	Mean (SE)	*n*	Mean (SE)		*n*	Mean (SE)	*n*	Mean (SE)	
Time from IHR to survey (months)	83	5.7 (0.3)	83	6.0 (0.3)	0.30	85	5.7 (0.3)	85	6.7 (0.3)	0.03
Pain										
Time from IHR to little or no pain (days)	74	15.5 (1.6)	79	14.0 (1.4)	0.17	76	15.5 (1.5)	75	18.2 (2.0)	0.33
Time from IHR to no Rx pain medications (days)	66	9.4 (1.5)	66	11.6 (1.7)	0.30	69	9.4 (1.4)	69	10.6 (1.2)	0.03
Activity level										
Time from IHR to resume activities (days)	79	18.0 (1.6)	79	118.0 (1.8)	0.60	81	18.0 (1.5)	78	20.2 (1.6)	0.34
Time from IHR to return to work (days)	60	17.8 (2.1)	54	17.9 (2.8)	0.34	64	17.0 (2.0)	44	21.7 (2.4)	0.08
Number of follow-up visits after IHR	83	1.6 (0.1)	83	1.5 (0.1)	0.51	85	1.6 (0.1)	85	1.8 (0.1)	0.08

*n* number of respondents, *IHR* inguinal hernia repair, *L* laparoscopic, *O* open, *R* robotic assisted, *Rx* prescription, *SE* standard error

The *p* values were calculated using Wilcoxon rank sum test

### Perceived physical activity limitations

Respondents recalled less activity disruption 1 week postoperatively after R-IHR compared to O-IHR (6.1 ± 0.3 vs. 7.3 ± 0.2, *p* < 0.01) but similar levels of activity disruption after R-IHR compared to L-IHR (6.0 ± 0.3 vs. 6.6 ± 0.27, *p* = 0.32). They reported returning to work at similar times postoperatively regardless of operative technique. At the time of the survey, respondents perceived the level of physical activity restrictions to be lower after R-IHR compared to O-IHR (1.4 ± 0.2 vs. 2.8 ± 0.4, *p* < 0.01) but similar between R-IHR and L-IHR (1.3 ± 0.2 vs 1.2 ± 0.2, *p* = 0.94). A summary of physical activity disruption appears in Tables 2 and 3.

**Table 3:** Respondent perceptions of postoperative groin pain, activity level, and overall satisfaction (propensity matched)

Measures	Robotic vs laparoscopic					Robotic vs open				
	R-IHR (*n* = 83)		L-IHR (*n* = 83)		*p* value	R-IHR (*n* = 85)		O-IHR (*n* = 85)		*p* value
	*n*	Mean (SE)	*n*	Mean (SE)		*n*	Mean (SE)	n	Mean (SE)	
Pain										
Rating of groin pain 1 week prior to IHR	83	5.4 (0.4)	83	5.8 (0.3)	0.49	85	5.6 (0.3)	85	5.8 (0.4)	0.68
Rating of groin pain 1 week after IHR	83	4.0 (0.3)	83	4.4 (0.3)	0.37	85	4.1 (0.3)	85	5.6 (0.3)	< 0.01
Rating of groin pain at time of survey	83	1.5 (0.3)	83	1.1 (0.2)	0.10	85	1.6 (0.3)	85	2.2 (0.3)	0.17
Activity level										
Rating of activity disruption 1 week after IHR	83	6.0 (0.3)	83	6.6 (0.3)	0.32	85	6.1 (0.3)	85	7.3 (0.2)	< 0.01
Rating of perceived physical activity restrictions at time of survey	83	1.3 (0.2)	83	1.2 (0.2)	0.94	85	1.4 (0.2)	85	2.8 (0.4)	< 0.01
Overall satisfaction										
Rating of satisfaction with IHR experience	83	8.8 (0.2)	83	8.9 (0.2)	0.61	85	8.6 (0.2)	85	8.3 (0.2)	0.10

Groin pain assessed the Numeric Pain Rating Scale, activity disruption assessed by modified Numeric Pain Scale, and perceived level of physical activity assessed by modified Numeric Pain Scale

*n* number of respondents, *IHR* inguinal hernia repair, *L* laparoscopic, *O* open, *R* robotic assisted, *Rx* prescription, *SE* standard error

The *p* values were calculated using Wilcoxon rank sum test

### Satisfaction

At survey completion, most respondents felt satisfied with their outcome regardless of operative approach (R-IHR, 81% vs. O-IHR, 77% and R-IHR, 83% vs L-IHR, 84%). As shown in Table 3, respondents noted moderately high satisfaction (rated ≥ 8 on a 0–10 scale) after R-IHR compared to O-IHR (8.6 ± 0.2 vs. 8.3 ± 0.2, *p* = 0.10) and L-IHR (8.8 ± 0.2 vs. 8.9 ± 0.2, *p* = 0.60).

### Factors influencing perceived postoperative groin pain and activity disruption

Respondents who previously underwent IHR recalled less groin pain at 1 week postoperatively after R-IHR or L-IHR compared to O-IHR (4.6 ± 0.7 and 4.8 ± 0.4 vs. 7.3 ± 0.2, *p* < 0.01, respectively), reported less need for prescription analgesia medication with R-IHR or L-IHR compared to O-IHR (8.1 ± 1.8 and 9.1 ± 1.5 vs. 16.9 ± 2.4, *p* = 0.02, respectively) and less physical activity disruption 1 week postoperatively after R-IHR compared to O-IHR and L-IHR (6.2 ± 0.6 vs. 7.2 ± 0.2 and 8.1 ± 0.2, *p* < 0.01, respectively). Respondents without a history of IHR reported a slightly shorter duration of APGP following R-IHR compared to O-IHR and L-IHR (3.8 ± 0.3 vs. 5.5 ± 0.2 and 4.9 ± 0.2, *p* < 0.01, respectively). Other patient perceptions and time variables stratified by history of IHR are summarized in Table 4.

**Table 4:** Stratification of patient perceptions and time variables by history of inguinal hernia repair (propensity matched)

	Respondents with prior IHR (*n* = 186)							Respondents without prior IHR (*n* = 340)						
	R-IHR		O-IHR		L-IHR		*p* value	R-IHR		O-IHR		L-IHR		*p* value
	*n*	Mean (SE)	*n*	Mean (SE)	*n*	Mean (SE)		*n*	Mean (SE)	*n*	Mean (SE)	*n*	Mean (SE)	
Pain														
Rating of groin pain 1 week after IHR	24	4.6 (0.7)	112	7.3 (0.2)	50	4.8 (0.4)	< 0.01	74	3.8 (0.3)	102	5.5 (0.2)	164	4.9 (0.2)	< 0.01
Time from IHR to little or no pain (days)	20	16.3 (4.2)	101	17.5 (2.0)	47	14.8 (2.3)	0.90	66	14.7 (1.3)	87	20.3 (1.8)	156	22.1 (4.1)	0.05
Time from IHR to no Rx pain meds (days)	16	8.1 (1.8)	97	16.9 (2.4)	40	9.1 (1.5)	0.02	64	9.0 (1.5)	87	11.2 (1.1)	142	12.6 (1.2)	0.04
Activity level														
Rating of limitations 1 week after IHR	24	6.2 (0.6)	112	8.1 (0.2)	50	7.2 (0.3)	< 0.01	74	6.2 (0.4)	102	7.2 (0.2)	164	6.9 (0.2)	0.10
Time from IHR to no limitations (days)	21	16.7 (2.6)	99	16.2 (1.5)	48	17.9 (2.5)	0.70	73	18.9 (1.8)	90	22.6 (1.5)	159	20.1 (1.3)	0.05
Time from IHR to return to work (days)	12	14.3 (3.0)	91	19.5 (2.1)	39	19.3 (3.5)	0.90	60	18.2 (2.4)	60	23.6 (2.0)	126	21.1 (1.7)	0.07

*n* number of respondents, *IHR* inguinal hernia repair, *L* laparoscopic, *O* open, *R* robotic assisted, *Rx* prescription, *SE* standard error

The *p* values were calculated using a Wilcoxon rank sum test

Respondents using prescription analgesia medications preoperatively recalled less APGP after R-IHR or L-IHR compared to O-IHR (4.7 ± 0.3 and 5.3 ± 0.4 vs. 7.6 ± 0.2, *p* < 0.01, respectively). These respondents also claimed a shorter disruption of physical activity 1 week postoperatively compared to O-IHR (7.1 ± 0.5 and 7.2 ± 0.3 days vs. 8.0 ± 0.2 days, *p* = 0.02, respectively). Similarly, respondents not using prescription analgesia medications preoperatively recalled less APGP and a shorter disruption of physical activity 1 week postoperatively after R-IHR compared to O-IHR and L-IHR (Table 5). Most respondents who recalled significant APGP (rated ≥ 8 on a 0–10 scale) also noted greater physical activity disruption regardless of operative approach (R-IHR 75%, O-IHR 82%, and L-IHR 85%).

**Table 5:** Stratification of patient perceptions and time variables by preoperative analgesic medication

	Respondents taking analgesic meds preop (*n* = 201)							Respondents not taking analgesic meds preop (*n* = 325)						
	R-IHR		O-IHR		L-IHR		*p* value	R-IHR		O-IHR		L-IHR		*p* value
	*n*	Mean (SE)	*n*	Mean (SE)	*n*	Mean (SE)		*n*	Mean (SE)	*n*	Mean (SE)	*n*	Mean (SE)	
Pain														
Rating of groin pain 1 week after IHR	27	4.7 (0.6)	113	7.6 (0.2)	61	5.3 (0.4)	< 0.01	71	3.8 (0.3)	101	5.2 (0.3)	153	4.7 (0.2)	< 0.01
Time from IHR to little or no pain (days)	22	13.6 (1.9)	101	17.7 (1.8)	57	20.1 (2.4)	0.30	64	15.6 (1.7)	87	20.1 (2.0)	146	20.5 (4.4)	0.40
Time from IHR to no Rx pain medications (days)	23	10.7 (1.5)	101	15.5 (1.4)	54	15.5 (2.0)	0.50	57	8.2 (1.7)	83	12.6 (2.5)	128	10.3 (1.1)	0.06
Activity level														
Rating of limitations 1 week after IHR	27	7.1 (0.5)	113	8.0 (0.2)	61	7.2 (0.3)	0.02	71	5.8 (0.4)	101	7.2 (0.2)	153	6.9 (0.2)	< 0.01
Time from IHR to no limitations (days)	24	18.8 (2.7)	99	17.2 (1.4)	57	21.9 (2.7)	0.30	70	18.2 (1.8)	90	21.5 (1.6)	150	18.7 (1.3)	0.20
Time from IHR to return to work (days)	22	16.8 (2.7)	97	20.7 (1.9)	50	22.8 (2.8)	0.60	50	17.8 (2.6)	54	21.8 (2.4)	115	19.8 (1.8)	0.30

*n* number of respondents, *meds* medications, *preop* preoperatively, *IHR* inguinal hernia repair, *L* laparoscopic, *O* open, *R* robotic assisted, *Rx* prescription, *SE* standard error

The *p* values were calculated using Wilcoxon rank sum test

## Discussion

There is a public health concern in the US over excessive prescribing and utilization of opioid medications for managing acute and chronic pain. Strategies to impact prescribing practices and minimize opioid use and/or abuse for primary IHR include using local anesthetic medications perioperatively as well as prescribing nonsteroidal anti-inflammatory drugs with minimal (or no) low-dose opioid postoperatively [14]. In addition to these strategies to minimize use of opioid analgesic medications for APGP, other important factors such as preoperative pain level, operative approach, inclusion of neurectomy, mesh choice, mesh fixation strategy, surgical site occurrence, and hernia recurrence may influence patients’ perception of groin pain and activity disruption after IHR [15–21]. This study investigated a specific factor, the operative approach, on a group of propensity-matched patients who self-reported their perception of groin pain and activity disruption after IHR.

Patient-reported outcomes of APGP and activity disruption are improved after minimally invasive procedures compared to open IHR [22]. The results from this study demonstrate that respondents who underwent R-IHR compared to O-IHR perceived less APGP, fewer physical activity limitations at 1 week and at least 6 months postoperatively, and shorter duration of prescription analgesic medication use. APGP, physical activity limitation, and duration of prescription analgesic use were statistically similar among respondents who underwent R-IHR and L-IHR. However, 18% fewer R-IHR compared to L-IHR respondents reported CPGP and R-IHR respondents used prescription analgesic medications for approximately 2 days less than L-IHR respondents. While these differences did not achieve statistical significance, the results may be clinically relevant given the desire to minimize the use of prescription pain medication and mitigate the risk of CPGP among patients eligible for minimally invasive IHR.

Evidence demonstrates that patients with groin hernia-related pain preoperatively are at increased risk of groin pain postoperatively, particularly CPGP [23]. Subgroup analysis of respondents taking versus not taking prescription analgesic medications for preoperative groin hernia-related pain showed several potentially relevant clinical differences. Respondents who did not require prescription analgesic medications preoperatively perceived significantly less APGP and noted earlier resolution of APGP after R-IHR compared to both O-IHR and L-IHR. Like trends noted among respondents with or without preoperative groin hernia-related pain, subgroup analysis of respondents who had or had not undergone prior IHR demonstrated potentially relevant clinical differences. Specifically, respondents who had prior IHR perceived a shorter duration of APGP after R-IHR compared to O-IHR and L-IHR. These findings suggest that compared to other approaches R-IHR may confer short-term benefits in terms of APGP to patients not taking groin hernia-related prescription analgesic medications preoperatively and/or those with previous inguinal hernia repair.

Respondents with preoperative groin pain requiring prescription analgesic medications perceived less APGP, noted earlier resolution of APGP, used prescription analgesic medications 2 days less, and noted fewer physical activity limitations after R-IHR compared to O-IHR but not L-IHR. Likewise, respondents with a history of prior IHR perceived similar outcomes after R-IHR and L-IHR. These data suggest that compared to O-IHR, patients taking groin hernia-related prescription analgesic medications preoperatively may benefit in terms of APGP from a minimally invasive IHR approach.

Evidence supports the idea that a minimally invasive approach to IHR benefits select groups of patients. A recent multi-institutional retrospective study of propensity-matched patients with obesity (body mass index ≥ 30 kg/m^2^) who underwent R-IHR (*n* = 95) or O-IHR (*n* = 93) found that those who underwent R-IHR had a 7.6% lower rate of post discharge to 30-day complications [24]. Another single-surgeon study that compared R-IHR (*n* = 118) and L-IHR (*n* = 157) demonstrated equivalent short-term outcomes despite appreciably more complex patients in the R-IHR cohort [25]. Other single-surgeon, retrospective studies demonstrate the potential for R-IHR to lower patients’ perception of APGP, shorten recovery room time, and lower the rate of self-reported CPGP compared to L-IHR [22, 26].

Limitations of this study are worthy of mention and include a low survey response rate as well as respondent selection bias, non-response bias, and recall bias (mitigated through use of propensity matching on demographics and clinical information). The survey was written to minimize leading bias but the questions were not validated as neutral by pretesting. Additionally, the convenience sample of patients may not reflect the opinions of patients throughout the US or globally. Another potential limitation is the fact that respondents were culled from two different sources—market research panels and surgical practices. Although most respondents were identified from market research panels (~ 75%), efforts were undertaken to recruit from throughout the continental US to limit geographic influence.

In summary, this survey study demonstrated that patient perceptions of pain and activity disruption differ by approach, suggesting a potential advantage for surgeons to consider a minimally invasive technique over open IHR. Further prospective studies are needed to determine long-term outcomes, including a better understanding into the manifestation of pain in daily life as well as patient perceptions of pain and quality of life associated with IHR.

## References

[1] Everhart J (ed) (1994) US Department of Health and Human Services, Public Health Service, National Institutes of Health, National Institute of Diabetes and Digestive and Kidney Diseases. Digestive diseases in the United States: epidemiology and impact. Washington, DC. NIH publication no. 94-1447

[2] Rutkow I (2003). Demographic and socioeconomic aspects of hernia repair in the United States in 2003. Surg Clin N Am.

[3] Nguyen D, Parviz A, Chen D (2016). Groin pain after inguinal hernia repair. Adv Surg.

[4] Cox T, Huntington C, Blair L (2016). Predictive modeling for chronic pain after ventral hernia repair. Am J Surg.

[5] Kehlet H, Jensen T, Woolf C (2006). Persistent postsurgical pain: risk factors and prevention. Lancet.

[6] Wu C, Rowlingson A, Partin AW (2005). Correlation of postoperative pain to quality of recover in the immediate postoperative period. Reg Anesth Pain Med.

[7] Bower A, Royce A (2016). The importance of postoperative quality of recovery: influences, assessment and clinical and prognostic implications. Can J Anesth.

[8] Chou R, Gordon DB, de Leon-Casasola OA (2016). Guidelines on the management of postoperative pain. J Pain.

[9] Earle D, Roth J, Saber A (2016). SAGES guidelines for laparoscopic ventral hernia repair. Surg Endosc.

[10] Ballecer C, Felix C, Prebil BE, Campanelli G (2017). Robotic transabdominal preperitoneal inguinal hernia repair. Inguinal hernia surgery.

[11] Tiwari M, Reynoso J, High R (2011). Safety, efficacy, and cost-effectiveness of common laparoscopic procedures. Surg Endosc.

[12] Arcerito M, Changchien E, Bernal O (2016). Robotic inguinal hernia repair: technique and early experience. Am Surg.

[13] McCaffery M, Pasero C (1999). Pain: clinical manual.

[14] Mylonas KS, Reinhorn M, Ott LR (2017). Patient-reported opioid analgesic requirements after elective inguinal hernia repair: a call for procedure-specific opioid-administration strategies. Surgery.

[15] Zwaans WAR, Verhagen T, Wouters L (2017). Groin pain characteristics and recurrence rates: three-year results of a randomized controlled trial comparing self-gripping Progrip mesh and sutured polypropylene mesh for open inguinal hernia repair. Ann Surg.

[16] Andresen K, Fenger AQ, Burcharth J (2017). Mesh fixation methods and chronic pain after transabdominal preperitoneal (TAPP) inguinal hernia surgery: a comparison between fibrin sealant and tacks. Surg Endosc.

[17] Gitelis ME, Patel L, Deasis F (2016). Laparoscopic totally extraperitoneal groin hernia repair and quality of life at 2-year follow-up. J Am Coll Surg.

[18] Wennergren JE, Plymale M, Davenport D (2016). Quality of life scores in laparoscopic preperitoneal inguinal hernia repair. Surg Endosc.

[19] Lange JF, Kaufmann R, Wijsmuller AR (2015). An international consensus algorithm for management of chronic postoperative inguinal pain. Hernia.

[20] Shah NS, Fullwood C, Siriwardena AK, Sheen AJ (2014). Mesh fixation at laparoscopic inguinal hernia repair: a meta-analysis comparing tissue glue and tack fixation. World J Surg.

[21] Zannoni M, Luzietti E, Viani L (2014). Wide resection of inguinal nerve versus simple section to prevent postoperative pain after prosthetic inguinal hernioplasty: our experience. World J Surg.

[22] Iraniha A, Peloquin J (2017). Long-term quality of life and outcomes following robotic assisted TAPP inguinal hernia repair. J Robot Surg.

[23] Olsson A, Sandblom G, Fränneby U (2017). Impact of postoperative complications on the risk for chronic groin pain after open inguinal hernia repair. Surgery.

[24] Kolachalam R, Dickens E, D’Amico L (2017). Early outcomes of robotic-assisted inguinal hernia repair in obese patients: a multi-institutional, retrospective study. Surg Endosc.

[25] Kudsi OY, McCarty JC, Paluvoi N, Mabardy AS (2017). Transition from laparoscopic totally extraperitoneal inguinal hernia repair to robotic transabdominal preperitoneal inguinal hernia repair: a retrospective review of a single surgeon’s experience. World J Surg.

[26] Waite KE, Herman MA, Doyle PJ (2016). Comparison of robotic versus laparoscopic transabdominal preperitoneal (TAPP) inguinal hernia repair. J Robot Surg.

