# Correction to: Patient perceptions of acute pain and activity disruption following inguinal hernia repair: a propensity-matched comparison of robotic-assisted, laparoscopic, and open approaches

**DOI:** 10.1007/s11701-018-0831-4

**Published:** 2018-06-18

**Authors:** James G. Bittner IV, Lawrence W. Cesnik, Thomas Kirwan, Laurie Wolf, Dongjing Guo

**Affiliations:** 1 Department of Surgery, Virginia Commonwealth University School of Medicine, PO Box 980519, Richmond, VA 23298 USA; 2 Market Research Department, Intuitive Surgical, Inc., Sunnyvale, CA USA; 3 Bruno and Ridgway Research Associates, Lawrenceville, NJ USA; 4 Department of Clinical Affairs, Intuitive Surgical, Inc., Sunnyvale, CA USA

## Correction to: Journal of Robotic Surgery 10.1007/s11701-018-0790-9

Unfortunately, the online published article has errors in Table 2. The number “118.0” found under the column “L-IHR” and row “Time from IHR to resume activities” should be corrected to 18.0.

The correct Table 2 is given below.

**Table 2:** Time variables related to postoperative groin pain and activity level (propensity matched)

Measures	Robotic vs laparoscopic					Robotic vs open				
	R-IHR (*n* = 83)		L-IHR (*n* = 83)		*p* value	R-IHR (*n* = 85)		O-IHR (*n* = 85)		*p* value
	*n*	Mean (SE)	*n*	Mean (SE)		*n*	Mean (SE)	*n*	Mean (SE)	
Time from IHR to survey (months)	83	5.7 (0.3)	83	6.0 (0.3)	0.30	85	5.7 (0.3)	85	6.7 (0.3)	0.03
Pain										
Time from IHR to little or no pain (days)	74	15.5 (1.6)	79	14.0 (1.4)	0.17	76	15.5 (1.5)	75	18.2 (2.0)	0.33
Time from IHR to no Rx pain medications (days)	66	9.4 (1.5)	66	11.6 (1.7)	0.30	69	9.4 (1.4)	69	10.6 (1.2)	0.03
Activity level										
Time from IHR to resume activities (days)	79	18.0 (1.6)	79	18.0 (1.8)	0.60	81	18.0 (1.5)	78	20.2 (1.6)	0.34
Time from IHR to return to work (days)	60	17.8 (2.1)	54	17.9 (2.8)	0.34	64	17.0 (2.0)	44	21.7 (2.4)	0.08
Number of follow-up visits after IHR	83	1.6 (0.1)	83	1.5 (0.1)	0.51	85	1.6 (0.1)	85	1.8 (0.1)	0.08

*n* number of respondents, *IHR* inguinal hernia repair, *L* laparoscopic, *O* open, *R* robotic assisted, *Rx* prescription, *SE* standard error

The *p* values were calculated using Wilcoxon rank sum test

